# Complete Genome Sequences of Three Leptospira mayottensis Strains from Tenrecs That Are Endemic in the Malagasy Region

**DOI:** 10.1128/MRA.01188-18

**Published:** 2018-10-18

**Authors:** Colette Cordonin, Céline Toty, Patrick Mavingui, Pablo Tortosa

**Affiliations:** a Unité Mixte de Recherche Processus Infectieux en Milieu Insulaire Tropical (UMR PIMIT), Université de La Réunion, INSERM 1187, CNRS 9192, IRD 249, Plateforme de recherche CYROI, Sainte-Clotilde, La Réunion, France; b Unité Mixte de Recherche Maladies Infectieuses et Vecteurs: Evolution, Génétique, Ecologie, Contrôle (UMR MIVEGEC), Université de Montpellier, UMR 5290 CNRS/IRD, UR 244 IRD, Montpellier, France; University of California, Riverside

## Abstract

Leptospirosis is a zoonosis caused by Leptospira, a diversified genus containing more than 10 pathogenic species. Tenrecs are small terrestrial mammals endemic in the Malagasy region and are known to be reservoirs of the recently described species Leptospira mayottensis. We report the complete genome sequences of three L. mayottensis strains isolated from two tenrec species.

## Abstract (precis)

Leptospirosis is a zoonosis caused by Leptospira, a diversified genus containing more than 10 pathogenic species. Tenrecs are small terrestrial mammals endemic in the Malagasy region and are known to be reservoirs of the recently described species Leptospira mayottensis.

## ANNOUNCEMENT

Leptospirosis is an environmental infectious disease caused by spirochetal bacteria belonging to the genus Leptospira. Humans usually get infected during recreational or work-related outdoor activities through contact with urine excreted by animal reservoirs. This zoonosis is estimated to cause more than one million human cases and 58,900 deaths each year (1). Disease incidence is highest on tropical islands, notably in the southwestern Indian Ocean, where investigations carried out under a One Health framework have revealed distinct transmission chains in the different islands (2–5), including the occurrence of Leptospira mayottensis, a pathogenic Leptospira species recently recognized as new to science (6). Representatives of L. mayottensis (formerly known as Leptospira borgpetersenii group B) were originally isolated from human acute cases (7) and later identified in tenrecs (2, 5, 8), a diversified family of mammals endemic to Madagascar (9, 10).

Three L. mayottensis strains were isolated in the field from two tenrec species, namely, Tenrec ecaudatus on Mayotte (MDI222 and MDI272) (2) and Microgale dobsoni on Madagascar (VS2413) (5). Frozen isolates were thawed in liquid Ellinghausen-McCullough-Johnson-Harris (EMJH) medium containing albumin fatty acid supplements (AFAS) (3), and they were further subcultured in EMJH supplemented with AFAS, 1% fetal calf serum, and 8% rabbit serum. DNA was extracted with the EZ1 virus minikit v.2.0 (Qiagen, Germany), and sequencing was performed on PacBio platforms. The sequence for VS2413 was assembled *de novo* with SMRT Analysis HGAP.2, those for MDI222 and MDI272 were assembled *de novo* using Canu 1.6, and contigs were circularized using AMOS v.3.1 (11).

Overall, genome sizes and structures (see Table 1) are close to those of previously published ones, with comparably low (≤40%) GC contents, two chromosomes, and, in the case of T. ecaudatus-borne strains, an additional plasmid (12).

**TABLE 1:** Genome architecture of *L. mayottensis* isolates^*a*^

Isolate	Source host	Sampling location	Chromosome length (bp)		Plasmid length (bp)	No. of reads	*N*_50_ length (bp)
			I	II			
MDI222	*Tenrec ecaudatus*	Mayotte	3,934,331 (not circularized)	330,241	111,328	44,976	20,779
MDI272	*Tenrec ecaudatus*	Mayotte	3,861,885	332,647	52,509	52,101	17,832
VS2413	*Microgale dobsoni*	Madagascar	3,787,562	300,219		55,595	21,656

a All but one replicon were successfully circularized.

Automatic annotation was performed with the NCBI Prokaryotic Genome Annotation Pipeline (13), which revealed that the VS2413, MDI272, and MDI222 genomes are composed of 3,893, 3,917, and 4,136 coding sequences, respectively.

Human leptospirosis on Mayotte is peculiar because it is associated with four distinct bacterial species, including L. mayottensis (14), and characterized by a relatively low fatality rate (0.9%) (15). The epidemiology is clearly distinct in the neighboring Seychelles and on Reunion Island, where L. interrogans is overwhelmingly dominant and fatality rates are higher (11.8% in the Seychelles and 3 to 5% on Reunion Island) (3, 4, 16). An attenuated virulence of Leptospira species prevailing on Mayotte, including L. mayottensis, may be at least in part responsible for this contrasted regional epidemiology. The genomes presented herein will accelerate comparative genomic approaches aimed at delineating the main genomic features involved in Leptospira virulence.

### Data availability.

Genome sequences were deposited in DDBJ/EMBL/GenBank under the accession numbers CP030142 and CP030143, CP030144 to CP030146, and CP030147 to CP030149 for strains VS2413, MDI222, and MDI272, respectively, as well as BioProject number PRJNA477299. Raw sequence data are available from the NCBI Sequence Read Archive (accession number SRP154442).
